# Efficacy and Mechanism of Panax Ginseng in Experimental Stroke

**DOI:** 10.3389/fnins.2019.00294

**Published:** 2019-04-24

**Authors:** Lei Liu, Gigi A. Anderson, Tyler G. Fernandez, Sylvain Doré

**Affiliations:** ^1^Department of Anesthesiology, Center for Translational Research in Neurodegenerative Disease and McKnight Brain Institute, University of Florida, Gainesville, FL, United States; ^2^Departments of Neurology, Psychiatry, Pharmaceutics, and Neuroscience, University of Florida, Gainesville, FL, United States

**Keywords:** ginsenosides, global cerebral ischemia, intracerebral hemorrhage, middle cerebral artery occlusion, permanent MCAO, subarachnoid hemorrhage, transient MCAO

## Abstract

Stroke is one of the leading causes of death and long-term disability worldwide. However, effective therapeutic approaches are still limited. The disruption of blood supply triggers complicated temporal and spatial events involving hemodynamic, biochemical, and neurophysiologic changes, eventually leading to pathological disturbance and diverse clinical symptoms. Ginseng (*Panax ginseng*), a popular herb distributed in East Asia, has been extensively used as medicinal and nutritional supplements for a variety of disorders worldwide. In recent years, ginseng has displayed attractive beneficial effects in distinct neurological disorders including stroke, involving multiple protective mechanisms. In this article, we reviewed the literature on ginseng studies in the experimental stroke field, particularly focusing on the *in vivo* evidence on the preventive or therapeutic efficacy and mechanisms of ginseng and ginsenosides in various stroke models of mice and rats. We also summarized the efficacy and underlying mechanisms of ginseng and ginsenosides on short- and long-term stroke outcomes.

## Introduction

Ginseng (*Panax ginseng C. A. Meyer*) has been extensively used as medicinal and nutritional supplements for a variety of disorders worldwide (Rastogi et al., 2014; Colzani et al., 2016). Asian ginseng has a history of herbal use over thousands of years, first described in the ancient Chinese pharmacopeia, Shen Nong Ben Cao Jing (300 BC−200 AD, also *Divine Farmer's Classic of Materia Medica*) (Unschuld, 1985; Yang and Wu, 2016). It is one of the most highly regarded herbs in the Orient used to promote health, general body vigor, and to prolong life span. The Greek word “*Panax*” originates from the word “panacea,” which means “cure all diseases,” and true to its name, ginseng has been proven to have a wide variety of medicinal uses, including benefits in cardiovascular disorders (Karmazyn et al., 2011; Sun et al., 2016; Kim, 2018), aging-related disorders (Bjorklund et al., 2018), and others (Sotaniemi et al., 1995; An et al., 2011; Shergis et al., 2014; Zhang et al., 2017; Arring et al., 2018). In recent years, preclinical and clinical studies revealed that ginseng displayed attractive beneficial effects in multiple neurological disorders like stroke, hypertension, cancer, and maintenance of hemostasis in the immune system, involving multiple protective mechanisms (Lee et al., 2009; Im and Nah, 2013; Rastogi et al., 2014; Gonzalez-Burgos et al., 2015; Ong et al., 2015; Oh and Kim, 2016; Wang et al., 2016b; Kim et al., 2018).

Stroke is a leading cause of death and long-term disability worldwide (Feigin et al., 2017; Benjamin et al., 2018); however, effective therapies are limited (Feigin et al., 2016). The disruption of blood supply triggers complicated temporal and spatial events involving hemodynamic, biochemical, and neurophysiologic changes, eventually leading to pathological disturbance and diverse clinical symptoms (Lo et al., 2003; Iadecola and Anrather, 2011; Annunziato et al., 2013; Bernhardt et al., 2018). The severity and dynamic progression of brain injury depend on the degree of cerebral blood flow (CBF) interruption, lesion volume and site, duration of stroke, and the coexisting complications (Shen and Duong, 2008; Sun et al., 2014b; Fu et al., 2015; Ward, 2017). Accumulated evidence shows that oxidative stress and inflammation play key roles in the pathophysiology of stroke (Iadecola and Anrather, 2011; Li et al., 2011a; Carbone et al., 2015; Fu et al., 2015). Although the ginseng remedy has been widely applied to improve cardiac health and circulation, their studies in the stroke field are still limited (Gan and Karmazyn, 2018; Kim, 2018). Over the last decade, much promising advancements were made in the therapeutic effects of ginseng or ginsenosides on experimental stroke brain injury.

In this article, we reviewed the literature on ginseng and ginsenosides studies in the experimental stroke field, particularly focusing on the *in vivo* evidence in diverse stroke models of mice and rats. We summarized the efficacy of ginseng and ginsenosides on short- and long-term stroke outcomes, as well as the underlying molecular and cellular mechanisms. This review provides current understanding of the pharmacological benefits of ginseng that contribute to stroke prevention and recovery.

## Panax Ginseng and Its Active Constituents

Two common products of ginseng are red ginseng, prepared by a process of steaming or heating, and dried white ginseng, prepared by air-drying after harvest (Wang et al., 2016a; He et al., 2018). Due to the presence of different active components, they have distinct pharmacodynamics profiles (Karmazyn et al., 2011). The major active components responsible for the pharmacological activities of ginseng are a group of unique triterpene glycosides or saponins called ginsenosides. The first attempt to isolate the active constituents of ginseng began many years ago, and the isolation of ginsenosides was started in 1963 (Shibata et al., 1963). To date, more than 150 ginsenosides have been isolated from ginseng, 40 of which have been found in *Panax ginseng* (Christensen, 2009).

**Figure 1:**
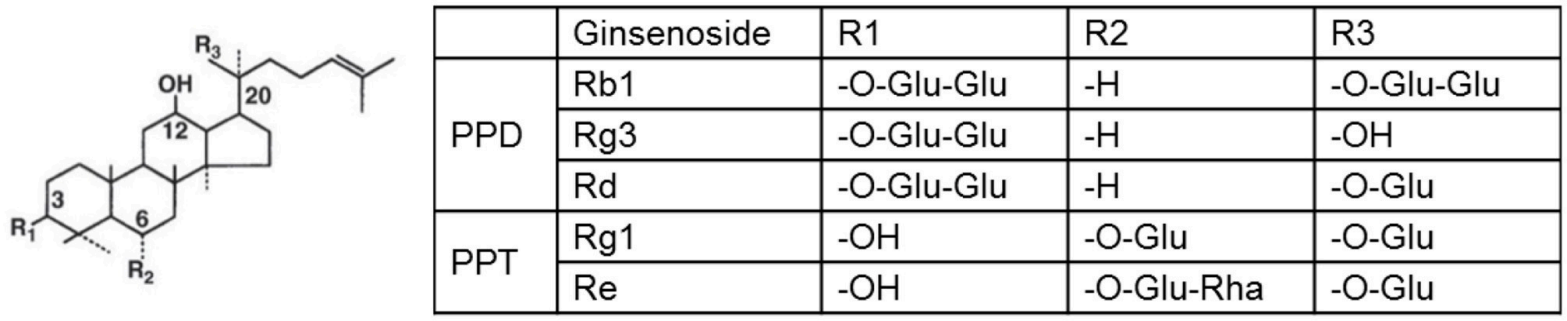
Chemical structures of most commonly studied ginsenosides. Glu, glucose; Rha, rhamnose.

Ginsenosides are divided into two different structural classes: (1) The 20(S)-protopanaxadiol (PPD) type that includes Ra1, Ra2, Ra3, Rb1, Rb2, Rb3, Rc, Rd, Rg3, Rh2, F2, and compound K; (2) The 20(S)-protopanaxatriol (PPT) type that includes Re, Rf, Rg1, Rg2, Rh1, and F1 (Baek et al., 2012). They share a four-ring hydrophobic steroid-like structure with sugar moieties, but differ in the carbohydrate moieties at C3, C6, and C20. Figure 1 shows the chemical structures of some of the most commonly studied ginsenosides. Quantitative and statistical analyses of the plasma indicate that PPD ginsenosides exhibit higher concentration and longer half-life than PPT ginsenosides (Zhang et al., 2014b). The peak concentrations of ginsenosides Rb1, Rb2/b3, Rc, Rd, Rg1, and Re are 55.32, 30.22, 21.42, 8.81, 7.15, 2.83 mg/l, while their mean values of half-lives are 18.41, 27.70, 21.86, 61.58, 15.26, and 2.46 h, respectively.

Intact ginsenosides are absorbed only through the intestines with a very low absorption rate at 1–3.7%. Most ginsenosides are metabolized in the stomach (acid hydrolysis) and/or intestine (bacterial hydrolysis) and transformed to other ginsenosides (Oh and Kim, 2016). For instance, ginsenoside Rb1 is processed by gastric acid/intestinal microorganisms into smaller molecules, such as Rd, F2, and compound K, and further into PPD. Similarly, ginsenoside Rg1 is converted into Rh1 and F1, and further into PPT, which is better absorbed in the gastrointestinal tract and therefore more bioactive than parent compounds. Collective evidences suggest that the metabolism and transformation of intact ginsenosides is a crucial process, influencing the bioavailability and potential health benefits of ginseng (Chen et al., 2008a).

## Stroke Models of Mice and Rats

Stroke can be classified into two types: ischemic stroke and hemorrhagic stroke. In ischemic stroke patients, the middle cerebral artery (MCA) is the artery most often blocked. Accordingly, focal cerebral ischemia models (permanent or transient) that aim at MCA territory have been most widely used (Dorr et al., 2007; Mehta et al., 2007). In contrast, global cerebral ischemia occurs when cerebral blood flow (CBF) is disrupted throughout whole brain. Hemorrhagic stroke is a devastating stroke subtype with a high mortality rate within 1 month; it mainly includes intracerebral and subarachnoid hemorrhage (Maclellan et al., 2010; Ma et al., 2011; Leclerc et al., 2018).

- Permanent focal cerebral ischemia (pdMCAO and pMCAO): The MCA can be occluded at distal or relatively proximal site; consequently they are termed as pdMCAO or pMCAO. Comparably, the pdMCAO model generates a reproducible ischemic lesion that is mainly restricted in cortex region and leads to definable sensorimotor deficits. Because it closely mimics human ischemic stroke, it serves as one of the most useful stroke models, allowing to assess long-term recovery with high survival rate (Doyle and Buckwalter, 2014). pMCAO can be produced by the intraluminal suture MCAO.
- Transient focal cerebral ischemia (tMCAO): The rodent MCAO with intraluminal suture is the most widely used animal stroke model, displaying reproducible MCA territory infarctions and allowing reperfusion by retracting the suture. Usually, MCAO generates ischemic infarct damage in the striatum, overlying frontal, temporal, parietal, and portions of cortex. Around 60 to 120 min of ischemia following MCAO is required to generate reproducible infarct volumes.
- Global cerebral ischemia (GCI): Due to cardiac arrest, GCI results in delayed neuronal death in the hippocampal CA1 region and subsequent cognitive decline (Traystman, 2003; Tu et al., 2015; Ostrowski et al., 2016). The four-vessel occlusion (4VO) model provides a method of reversible forebrain cerebral ischemia-reperfusion, whereas the two-vessel (2VO) model was developed to characterize the incomplete ischemia (Traystman, 2003).
- Cerebral hypoxia-ischemia (HI): HI is a transient unilateral cerebral ischemia model, which produces reproducible brain lesion in the ipsilateral hemisphere (Liu et al., 2019). Following the occlusion of one side of the common carotid artery and after a short recovery, the animal will be exposed to systemic hypoxia for no more than 1h.
- Intracerebral hemorrhage (ICH): ICH is a most devastating type of stroke without effective therapies. Two available models are used to mimic spontaneous intracerebral bleeding, either by the stereotactic injection of autologous blood or collagenase (Maclellan et al., 2010; Ma et al., 2016; Ahmad et al., 2017). Till now, no ginseng study has been performed in the ICH model.
- Subarachnoid hemorrhage (SAH): SAH claims one of the highest rates of mortalities and morbidities. None of therapeutic options has effectively to reduce mortality rate in a clinical setting. Rodent models have been predominantly made by approaches involving intravascular perforation of a vessel in the circle of Willis or direct injection of blood into the cisterna magna or prechiasmatic cistern (Leclerc et al., 2018).

### Therapeutic Effects of Panax Ginseng on Stroke Outcomes: The *in vivo* Evidence

Multiple administration strategies of ginseng have been employed in the experimental stroke studies, including mice or rats with different genetic backgrounds, pre-treatment or post-treatment, administration routes, dosage range and duration, and various histological and neurobehavioral stroke outcomes. Infarct volume is designed to evaluate the temporal evolution of stroke damage that can be easily measured with different techniques. Neurobehavioral assessment is an essential measure of stroke outcome since functional recovery is universally used as a primary endpoint in clinical trials. Both histological and neurobehavioral measurements are considered as pivotal components for examining efficacy of potential therapeutics in the translational stroke research field. Here, we outlined the short-term (usually referring to the acute stage of recovery following stroke, about 1–3d) and long-term (usually referring to 3d to weeks or months following stroke) effects of ginseng and ginsenosides on stroke outcomes. Table 1 summarized the details of these studies.

**Table 1:** The effects of various ginseng extracts on stroke outcomes in rodent stroke models.

**Species**	**Genetic background**	**Type of ginseng extracts**	**Dosage/Administration route**	**Time of termination**	**Brain lesion /Edema**	**Neurobehavioral deficits**	**Authors/year**
**CEREBRAL ISCHEMIA**							
**Permanent Distal Middle Cerebral Artery Occlusion (pdMCAO)**							
Mouse	C57BL/6	Red ginseng(KRG)	• Pre;100 mg/kg/d; gavage • For 7d	28d	• Infarct volume (1d, 3d ↓) • Brain edema (1, 3d) ↓	(pre, 1, 3, 7, 14, 21, 28d for all tests) • Open field • Cylinder (3, 7, 28d) ↓ • Corner (3, 7, 14, 28d) ↓	Liu et al., 2018b
Rat	SHR-SP (spontaneous hypertensive rats-stroke prone)	Ginsenoside Rb1	• Post; 60 ul (6 or 60 μg/60 ul/d); i.v.; immediately after MCAO; infusion for 4 wks • 60 μl (6, 60, 3,000, or 12,000 μg/60 μl/d); i.v.; at 2 h after MCAO; infusion for 1d	2, 4 wks	• Infarct size ↓ • (28d or delayed treatment 1d; 6 or 60 μg/60 ul/d)	• Water maze (2, 4 wks) ↓	Zhang et al., 2006
Rat	SH-SP	Ginsenoside Rb1	• (0.006 to 6.0) μg/d; i.c.v.; started 2 h before MCAO; infusion for 4 wks; and (0.06, 0.6, 6.0) μg/d; i.c.v.; started immediately after MCAO • Infusion for 4 weeks	2, 4 wks	• Infarct size ↓ 32d; Rb1 (0.6 μg/d)	• Water maze (2, 4 wks); Rb1 (0.6 μg/d) ↓ • Inclined Screen (muscle strength, 2, 4 wks) • Rotarod (2, 4 wks)	Zhang et al., 1998
**Permanent Middle Cerebral Artery Occlusion (pMCAO)**							
Rat	SD	Ginsenosides Rb1 and Rg1	pMCAO • Pre; Rb1 (10, 20 and 40 mg/kg); Rg1 (40 mg/kg); i.v.; 30 min before MCAO MCAO (2 h) • Pre/Post; Rb1 (10, 20, and 40 mg/kg); Rg1 (40 mg/kg); i.v.; 30 min before MCAO or immediately after MCAO	24 h	• Infarct size (24 h) • pMCAO: Rb1 (40 mg/kg) ↓; • tMCAO (2 h): Rb1 (10, 20, and 40 mg/kg) ↓ • Brain edema (24 h) • pMCAO: Rb1 (40 mg/kg) ↓ • tMCAO (2 h): Rb1 (10, 20, and 40 mg/kg) ↓Rg1 (40 mg/kg) ↓	pMCAO: • Neurologic deficits score (24 h) Rb1 (40 mg/kg) ↓ • MCAO (2 h) • Neurologic deficits score (24 h) Rb1 (10, 20 and 40 mg/kg) ↓	Zhang and Liu, 1996
Rat	SD	Ginsenoside Rd	• Pre; dose-response study: 1–50 mg/kg; i.p.; 30 min before MCAO • Post; therapeutic window study: 50 mg/kg; i.p.; at 0, 2, 4, or 8 h after the reperfusion for transient ischemia or after the onset of artery occlusion for permanent ischemia	24 h for pMCAO; 42d for tMCAO (2 h)	dose-response study • Infarct volume (1,3,7d; 10 and 50 mg/kg) ↓ • Brain edema (24 h; 10 and 50 mg/kg) ↓ therapeutic window study • Infarct volume (24 h; pMCAO; 2 h or 4 h treatment, not 8 h) ↓ • Infarct volume (1,3,7d; tMCAO; 2 or 4 h treatment, not 8 h) ↓	dose-response study (pre, 1, 3, 7, 14, 21, 28, 42d) • Modified neurological severity score (10,50 mg/kg) ↓ • Modified sticky-tape test (10,50 mg/kg) ↑ • Corner (50 mg/kg) ↓therapeutic window study (pre, 1, 3, 7, 14, 21, 28, 42d) • Modified neurological severity score (2 or 4 h treatment) ↓ • Modified sticky-tape test (2 or 4 h treatment) ↑ • Corner (2 or 4 h treatment) ↓	Ye et al., 2011a
Rat	Wistar	Ginseng total saponins (GTS)	• Pre/Post; 25 mg/kg/d; i.p.; start 3d before MCAO; twice daily • For 1,3,7,14d	1, 3, 7, 14d	• ND	• Neurological deficits score (14d) ↓	Zheng et al., 2011
**Transient Middle Cerebral Ischemia (MCAO, 0.5 h)**							
Mouse	C57BL/6	Compound K	• Pre; 30 mg/kg; i.p.;	3d	• Infarct volume (3d; total, cortex, but not striatum) ↓	• ND	Park et al., 2012
**Transient Middle Cerebral Ischemia (MCAO, 1 h)**							
Mouse	C57BL/6	Red ginseng (KRG)	• Pre; 360 mg/kg; i.p.; before MCAO • For 14d	4 and 24 h,1, 3, 7, 14d	• Infarct volume (24 h) ↓	• Rotarod (3, 7d) ↑	Cheon et al., 2013
Mouse	C57BL/6J	Ginsenoside Rb1	• Pre; 0.5, 1, 5 or 10 mg/kg, respectively; oral gavage; every 3d • From 1 year till 2 years old	1, 2, 3, 7d	• Infarction volume (48 h; 1, 5, 10 mg/kg) or (1, 3, 7d; 5 mg/kg) ↓ • Brain edema (48 h; 1, 5, 10 mg/kg) or (1, 3, 7d; 5 mg/kg) ↓	• Neurological Bederson score (48 h; 1, 5, 10 mg/kg) or (1, 3, 7d; 5 mg/kg) ↓	Dong et al., 2017
Mouse	ICR	Ginsenoside Rb1	• Post; 5, 20 or 40 mg/kg; i.p.; after 3 h reperfusion	24, 48 h	• Infarct volume (48h; 20 and 40 mg/kg) ↓ • Brain edema (48 h; 20 and 40 mg/kg) ↓	• Neurological behavior deficits (5 point test; 48 h; 20 and 40 mg/kg) ↓	Chen et al., 2015
Mouse	C57BL/6	Ginsenoside Rd	• Pre (dose-response study); 0.1, 1, 10, 50, and 200 mg/kg; i.p.; 30 min before MCAO onset; once • Post (therapeutic window study); 50 mg/kg; i.p.; 2, 4, 6, or 10 h after the onset of MCAO • Pre; 50 mg/kg; i.p.; 30 min before MCAO; once • Post; 50 mg/kg/d; i.p.; immediately after reperfusion till 7d	1, 14d	• dose-response study: • Infarct Volume (1d; 10 and 50 mg/kg) ↓ • therapeutic window study: Infarct Volume (1d; treatment at 2 or 4 h after reperfusion) ↓ • Pre: Infarct volume (14d; 50 mg/kg) ↓ • Post: no difference from pre	dose-response study: • NDS on a scale of 0-12 (1d; 10 and 50 mg/kg) ↓; NDS on a scale of 3-18 (1d; 10 and 50 mg/kg) ↑therapeutic window study: • NDS on a scale of 0-12 (1d; treatment at 2 h) ↓; NDS on a scale of 3–18 (1d; treatment at 2 or 4 h) ↑ • Pre: NDS on a scale of 0–12 (14d) ↓ NDS on a scale of 3–18 (14d) ↑ • Post: no difference from pre	Ye et al., 2011b
Mouse	BALB/c	Ginsenoside Rg1	• Post; 20 or 40 mg/kg; i.p.; 0.5 h after ischemia and 12 h after reperfusion	24 h	• Infarct volume (24 h; 40 mg/kg) ↓ • Brain edema (24 h; 40 mg/kg) ↓	• Neurological deficits score (24 h; 40 mg/kg) ↓	Sun et al., 2014a
Rat	SD	Ginsenoside Rg2	• Post; 2.5, 5, and 10 mg/kg; i.v.; 15 min before and 24 h after reperfusion • Twice	48 h	• Infarct volume (2.5 mg/kg) ↓	• Neurological deficits score (2.5, 5 and 10 mg/kg) ↓ • Y-maze ↓	Zhang et al., 2008a
**Transient Middle Cerebral Ischemia (MCAO, 1.5 h)**							
Rat	SD	Ginsenoside Rb1	• Post; 1.25 or 12.5 mg/kg; intranasal; right after MCAO • Once	24 h	• Infarct volume (24 h) ↓ • Nissl-positive neurons (24 h) ↑	• ND	Lu et al., 2011
Rat	SD	Ginsenoside Rd	• Post; 1, 2.5, and 5 mg/kg/d, i.p. • From 1d to 3d after MCAO	7d	• Infarct volume (7d; 5 and 2.5 mg/kg) ↓	• Neurological deficits score (24 h and 7d; 5 mg/kg) ↓	Liu et al., 2015
Rat	SD	Ginsenoside Rg1	• Post; 30, 60 mg/kg; i.p.; 0 and 6 h after reperfusion	24, 72 h	• Brain edema (24 h; 60 mg/kg) ↓	• Longa's Neurological deficits score (24 h, 72 h; 60 mg/kg) ↓	Li et al., 2017b
Rat	SD	Ginseng extracts	• Post; 200 mg/kg; orally after reperfusion; once daily • For a week	1, 3, 7, 10, and 15d	• Infarct volume (15d) ↓	• Rotarod (3d) ↓	Park et al., 2010
**Transient Middle Cerebral Ischemia (MCAO, 2 h)**							
Mouse	C57BL/6J	Ginsenoside Rg1	• Pre; 20,40 mg/kg/d; gavage • For 7d	24 h	• Infarct volume (40 mg/kg; 24 h) ↓	• Neurological deficits score (40 mg/kg; 24 h) ↓	Wang et al., 2018a
Rat	SD	Red ginseng (KRG)	• Post; 100 mg/kg/d, orally; after the onset of reperfusion; once daily • For 7d	1, 3, 7d	• ND	• Modified neurological severity score (3, 7d) ↓ • Corner (3, 7d) ↓	Ban et al., 2012
Rat	SD	Red ginseng (KRG)	• Post; 100 mg/kg/d; orally after reperfusion	1, 3, 7d	• Infarct volume (7d) ↓	• Modified neurologic severity score (1, 3, 7d) ↓ • Corner (1, 3, 7d) ↓	Lee et al., 2011
Rat	SD	Black ginseng (produced from red ginseng)	• Post; 100 or 400 mg/kg; p.o.; after MCAO; daily for 2 wks	2 wks	• Cresyl violet stained neurons in hippocampus ↓	• Morris water maze (2nd wk)	Park et al., 2011
Rat	SD	Fermented red ginseng	• Post; 100 mg/kg; orally; promptly prior to reperfusion • Once	22 h	• Infarct volume (22 h)	• ND	Bae et al., 2004
Rat	SD	Ginsenoside Rb1	• Pre; 12.5 mg/kg/d; Intranasal administration • For 7d	6, 12, 24,72 h	• Infarct Volume (24 h) ↓	• Modified neurological severity score (72 h) ↓	Zhu et al., 2012
Rat	SD	Ginsenoside Rb1	• 40 mg/kg/d; i.p.; start 3d before MCAO; once daily • Till the animals were sacrificed	6 h, 1, 3, and 7d	• Infarct volume (6 h, 1, 3, and 7d) ↓	• Neurological deficits score (6 h, 1, 3 and 7d) ↓	Li et al., 2018b
Rat	SD	Ginsenoside Rb1	• Post; 20, 40, and 80 mg/kg/d; i.p.; start immediately after ischemia • Once	24 h	• Infarct volume (40 and 80 mg/kg) ↓	• Neurologic deficits (24 h; 40 and 80 mg/kg) ↓	Liu et al., 2013
Rat	Wistar	Ginsenoside Rb1	• Post; 50, 100 and 200 mg/kg/d; i.v.; after ischemia	24 h	• Infarct volume (24 h; 50, 100, and 200 mg/kg) ↓	• Neurological deficits score (24 h; 100 and 200 mg/kg) ↓	Liu et al., 2018a
Rat	Wistar	Ginsenoside Rb1	• Post; 40 mg/kg; i.p.; Immediately after the onset of reperfusion • Once	3 h, 12 h, 1, 2, 3, 5, and 10d	• ND	• Modified Neurological severity score (3, 5d) ↓	Gao et al., 2010
Rat	SD	Ginsenoside Rd	• Pre; 0.1, 1, 10, 50, 200 mg/kg, i.p. 30 min before MCAO • Once	1, 3, 7, 14, 21, 28 and 42d	• Infarct size (1d; 10 and 50 mg/kg) ↓	• Modified neurological severity score (14, 21, 28 and 42d; 10 and 50 mg/kg) ↓	Ye et al., 2011c
Rat	SD	Ginsenoside Rd	• Pre; 50 mg/kg; i.p.; 30 min before MCAO • Once	1,14d	• Infarct volume (1, 14d) ↓	• Belayev's neurological score (1, 14d) ↓ • Garcia's neurological score (1, 14d) ↑	Ye et al., 2011d
Rat	SD	Ginsenoside Rd	• Post; 50 mg/kg; i.p.; at 0, 2, 4, 8 h after reperfusion • pMCAO 24 h • tMCAO 2 h, reperfusion 42d	1, 3, 7d	• Infarct volume (1, 3, and 7d) • Brain edema(1d)	pre, d1, 3, 7, 14, 21, 28, and 42d • Modified neurological severity score • Modified sticky-tape • Corner	Ye et al., 2011a
Rat	SD	Ginsenoside Rd	• Pre/Post; 30 mg/kg; i.p.; 1 h before MCAO + 10 mg/kg/d after MCAO	1, 7, 14, 26–32d	• Infarct volume (24 h) ↓	• Novel object recognition (26–32d) ↑ • Morris water maze (26–32d) ↓	Zhang et al., 2014a
Rat	SD	Ginsenoside Rd	• Pre/Post; 30 mg/kg 1 h before MCAO; + 10 mg/kg/d after MCAO • For 7d	2 h, 8 h, 24 h, 2–7d	• ND	• Zea-Longa neurological deficits score (3–7d) ↓	Yang et al., 2016
Rat	SD	Ginsenoside Rd	• Pre/Post; 50 mg/kg; i.p.; 30 min before MCAO or immediately after MCAO • Single dose	2, 12, 24 h	• Infarct volume (24 h) ↓	• Neurological deficits (Bederson's scoring system; 24 h) ↓	Xie et al., 2016
Rat	SD	20(R)-Ginsenoside Rg3	• Pre; 5, 10, and 20 mg/kg; i.p.; twice daily before MCAO	24 h	• Infarct volume ↓ (24 h; 10 and 20 mg/kg)	• Neurological deficits score (24 h; 10 and 20 mg/kg) ↓	He et al., 2012
Rat	SD	Ginsenoside Re	• Pre; 5 or 10 or 20 mg/kg/d; p.o. • For 7d	2, 24 h	• ND	• Neurological deficits (5 point; 24 h)	Chen et al., 2008b
Rat	SD	Ginsenoside Rg1	• Pre; 20, 40, 60 mg/kg; i.v.; 1 h before MCAO	24 h	• ND	• Neurological deficits score (24 h) ↓	Yang et al., 2015b
Rat	SD	Ginsenoside Rg1	• Post; 30, 60 mg/kg/d; i.v.; after 2 h reperfusion; twice daily • For 3d	1, 3d	• Infarct volume (3d) ↓	• Neurological deficits score (60 mg/kg on 1d, 30 and 60 mg/kg on 3d) ↓	Lin et al., 2015
Rat	SD	Ginsenoside Rg1	• 20 mg/kg; i.p.; 1 h before MCAO and repeated each 12 h • Till each experiment was completed	2, 24 h	• Cortical damage size (4 h, 1, 2, and 5d) ↓; • Nissl stained neurons in cortex (24 h) ↑	• Neurological deficits score (24 h) ↓	Zhang et al., 2008b
Rat	SD	Ginsenoside Rg1	• 20 mg/kg; i.p.; started 3d before MCAO; twice daily • Till the animals were killed;	6 h, 1, 3, 7, and 14d	• ND	• Neurological deficits score (6 h, 1, 3, 7, and 14d) ↓	Zhou et al., 2014
Rat	SD	Ginsenoside Rg1	• 20 mg/kg; i.p.; started 3d before MCAO; twice daily • Till the animals were sacrificed	6 h, 1, 3, 7, and 14d	• Infarct volume (3d) ↓	• Neurological deficits score (Zea-Longa; 1, 3, 7, and 14d) ↓	Xie et al., 2015
Rat	SD	Ginsenoside Rh2	• Post; 100 mg/kg; orally.; immediately prior to reperfusion	22 h	• Infarct volume (22 h) ↓	• ND	Park et al., 2004
**Global Cerebral Ischemia (GCI)**							
Rat (2VO)	Wistar	Ginsenoside Rb1	• Pre; 20 or 40 mg/kg; i.v.; 15 min before ischemia	24, 72 h	• CA1 neuronal death ↓	• ND	Luo et al., 2014
Rat (4VO)	Wistar	Panax Ginseng extracts	• Post; 100, 200, 500, 1,000 mg/kg; i.p.; two injections at 0 and 90 min after occlusion	7d	• CA1 Neuronal death ↓	• ND	Kim et al., 2009
Rat (4VO)	SD	Ginsenosides Rb + Ro	• Pre; 100 mg/kg; i.v.; 30 min before 4-vessel occlusion	1 h	• Brain edema (1 h) ↓	• ND	Chu and Chen, 1990
**Hypoxia-Ischemia (HI)**							
Mouse	C57BL/6	Red ginseng (KRG)	• Pre;100 mg/kg/d; gavage • For 7d	6 h, 1 and 7d	• Neuronal intensity (6 h) ↓ • Infarct volume (6 h, 1d, 7d) ↓ • Brain edema (6 h, 1d, 7d) ↓	• (pre, 6 h, 1, 3, and 7d) • Neurological deficits score (6 h, 1, 3, and 7d) ↓ • Open field (3, 7d ↓) • Cylinder (3, 7, 28d) ↓ • Corner (7d) ↓	Liu et al., 2019
**INTRACEREBRAL HEMORRHAGE (ICH)**							
NA							
**SUBARACHNOID HEMORRHAGE (SAH)**							
Rat	SD	Ginsenoside Rb1	• Post; 20 mg/kg; via vena caudalis; 30 min after the first SAH • Followed by additional 7d	6 and 24 h after the 1st SAH; 6, 24, 48, 72, 96, and 120 h after the 2nd SAH	• Brain edema (24 h after the second SAH) ↓ • Arterial vasospasm (120 h after the second SAH)	• Spontaneous activity score (96 h after the second SAH) ↓	Li et al., 2011b
Rat	NA	Ginsenoside Rb1	• 5 or 20 mg/kg	NA	• Cerebral vasospasm; • Brain edema	• Neurological deficits	Li et al., 2010

*MCAO, middle cerebral artery; pdMCAO, permanent distal middle cerebral artery occlusion; pMCAO, permanent (proximal end of) middle cerebral artery occlusion that is generated by the intraluminal suture MCAO; Pre, pretreatment; Post, posttreatment; i.p., intraperitoneal; i.v., intravenous; i.c.v., intracerebroventricular; The changes in brain lesion/edema and neurobehavioral deficits (↑ or ↓, exacerbated or attenuated stroke outcomes at indicated time point; no label at indicated time point, no significant difference); h, hour; d, day; wk, week; NA, no answer; ND, non-discussed*.

### Red Ginseng

The standard extracts of red ginseng (such as Korean red ginseng, KRG) are manufacture by the traditional preparation method (by a steaming or heating process) and contain most of the primary effective components, coordinately controlling the pharmacological efficacy in the body (Lee et al., 2015; Wang et al., 2016a). Many are converted from the major ginsenosides Rb1, Rb2, Rc, Rd, Rg1, and Re (Lee et al., 2015). The therapeutic efficacy of KRG on ischemic brain damage, at the dosage of 100–360 mg/kg/d for 7–14d, has been revealed in permanent and transient cerebral ischemia models by several groups. In the pdMCAO mouse model, KRG pretreatment prevented the acute enlargement of ischemic brain lesion (36.37 ± 7.45% on d3) and the definable sensorimotor deficits indicated by optimized cylinder and corner tests, and such functional benefits extended over 28d (Liu et al., 2018b). KRG pretreatment also reduced the infarct volume at 24 h and improved the coordinated motor deficits, indicated by the rotarod test, at 3 and 7d after MCAO (1 h) (Cheon et al., 2013). Recently, it was reported that pretreatment with Ginseng elicited robust neuroprotection against the deterioration of acute cerebral hypoxia-ischemia damage in an Nrf2-dependent manner, evidenced by the reductions of neurological deficits and brain infarction and edema at 6 h, 1 and 3d after HI (Liu et al., 2019). Such beneficial outcomes could be associated with the enhanced expression levels of Nrf2 target antioxidant proteins and anti-inflammation mediators. Meanwhile, KRG post-treated rats showed significant improvement in the neurological deficits for 7d indicated by the modified neurological deficits score (NDS) and corner test, as well as the infarct volume at 7d, following ischemia-reperfusion injury after MCAO (2 h) (Lee et al., 2011; Ban et al., 2012). In addition, ethanolic P. ginseng extracts post-treatment was reported to reduce rat hippocampal CA1 neuronal death 7d after global cerebral ischemic injury (Kim et al., 2009), further supporting the beneficial role of KRG in ischemic stroke.

### Ginsenoside Rb1

Ginsenoside Rb1 (Rb1) is a representative component of *Panax genus*, including *Panax ginseng* (Asian ginseng), *Panax quinquefolius* (American ginseng), and *Panax notoginseng* (Ahmed et al., 2016), which has exhibited potent efficacy on cardiovascular disorders like myocardial Ischemia-reperfusion Injury (Zheng et al., 2017). In pdMCAO rats with stroke-prone spontaneous hypertension, Rb1 pretreatment by intravenous infusion ameliorated ischemia-induced place navigation disability at 2 and 4 weeks evidenced by the water maze test, reduced muscle strength deficit in the inclined screen test, impaired coordinated four-leg movements function in the rotarod test, and decreased the volume of the cortical infarct lesion at 28 and 32d after ischemia (Zhang et al., 1998, 2006). In pMCAO rats, Rb1 pretreatment reduced acute ischemic brain damage in infarct volume and overall neurological deficits 24 h after ischemia (Zhang and Liu, 1996). In MCAO (1–2 h) rats, Rb1 pre- or post-treatment significantly reduced acute brain lesion, evidenced by infarct volume at 24 h (Lu et al., 2011; Zhu et al., 2012) or 48 h (Chen et al., 2015; Dong et al., 2017), brain edema (Dong et al., 2017) at 48 h, and neurobehavioral deficits indicted by the overall neurological deficits score at 48 h (Chen et al., 2015; Dong et al., 2017), 72 h (Gao et al., 2010; Zhu et al., 2012), and 5d (Gao et al., 2010) after reperfusion onset. In GCI (2VO) rats, Rb1 pretreatment protected against hippocampal CA1 neuronal death at the acute stage of ischemia (Luo et al., 2014). Besides its favorable role in ischemic stroke, Rb1 also exhibited extensive neuroprotection in subarachnoid hemorrhage brain damage. Rb1 treatment dramatically reduced brain edema, cerebral vasospasm, and neurological deficits including spontaneous activity (Li et al., 2010, 2011b), indicating the extensive benefits to stroke outcomes.

### Ginsenoside Rg1

Ginsenosides Rb1 and Rg1 (Rg1) are the most abundant ginsenosides in ginseng roots, exhibiting pharmacological properties in multiple neurological conditions (Gao et al., 2017b; Song et al., 2017; Mohanan et al., 2018). Multiple studies have revealed the preventive and therapeutic efficacy of Rg1 on acute ischemia-reperfusion brain damage and long-term recovery in MCAO (1–2 h) of mice and rats. Rb1 pre- or post-treatment reduced the infarct volume at 24 h (Sun et al., 2014a; Li et al., 2017b; Wang et al., 2018a) and 3d (Lin et al., 2015) and brain edema at 24 h, and attenuated overall neurological deficits at 6 h (Zhou et al., 2014), 24 h (Zhang et al., 2008b; Sun et al., 2014a; Zhou et al., 2014; Lin et al., 2015; Xie et al., 2015; Yang et al., 2015b; Li et al., 2017b; Wang et al., 2018a) and 3d (Zhou et al., 2014; Xie et al., 2015) following MCAO (1–2 h). The neurobehavioral protection was also observed at late stage of stroke, evidenced by the reduced neurological deficits at 7 and 14d after MCAO (1–2 h) (Zhou et al., 2014; Xie et al., 2015).

### Ginsenoside Rd

Similar as the ginseng extracts above, ginsenoside Rd (Rd) is another important ingredient of ginsenosides and widely investigated in the stroke field (Ye et al., 2013; Nabavi et al., 2015). In pdMCAO model mice, either pre-treatment or post-treatment of Rd prevented acute ischemic brain injury and promoted the long-term histological and neurobehavioral recovery, evidenced by the reduction of infarct volume at 1, 3, and 7d and neurological deficits score, sticky-tape test, and corner test over 42d after ischemia (Ye et al., 2011a). This benefit was also observed in ischemia-reperfusion rodent models. In MCAO (1.5 h) model rats, Rd post-treatment exhibited sustained neuroprotection against ischemic brain damage, indicated by the reduced neurological deficits at 1 and 7d and infarct volume at 7d after the onset of reperfusion (Liu et al., 2015). In MCAO (2 h), several studies showed that Rd treatment alleviated ischemia-reperfusion induced infarct volume at 24 h (Ye et al., 2011a,c,d; Zhang et al., 2014a; Xie et al., 2016), 3d, 7d (Ye et al., 2011a), and 14d (Ye et al., 2011d), and reduced overall neurological deficits at 1–42d (Ye et al., 2011a,c,d; Zhang et al., 2014a; Xie et al., 2016; Yang et al., 2016).

### Ginsenoside Rg3

Ginsenoside Rg3 (Rg3) is abundantly present in red ginseng preparation, which is highly known for its anticancer effects (Sun et al., 2017; Mohanan et al., 2018). A report showed that Rg3 pretreatment reduced ischemia-reperfusion injury, indicated by reduced infarct volume and overall neurological deficits score at 24 h after MCAO (2 h) (He et al., 2012).

### Ginsenoside Re

Ginsenoside Re (Re) is a major ginsenoside and important ingredient in ginseng leaf, berry, and root, exhibiting multiple pharmacological activities via different mechanisms (Peng et al., 2012). Re protected rats against acute brain lesion, indicated by the reduction of infarct volume at 24 h after MCAO (2 h) (Chen et al., 2008b).

### Ginsenoside Rh2

Ginsenoside Rh2 (Rh2), an important ginsenoside (Smith et al., 2014), was reported to reduce the acute ischemia-reperfusion damage indicated by reduced infarct volume at 22 h after MCAO (2 h) (Park et al., 2004).

### Compound K

Compound K is one of the major metabolites of ginseng, exhibiting a variety of pharmacological activities, including anti-inflammatory, antitumor, and other effects (Shin et al., 2015; Yang et al., 2015a). Compound K pretreatment significantly reduced the infarct volume (hemisphere, cortex, but not striatum) of ischemic brain after MCAO (0.5 h) (Park et al., 2012).

### Black Ginseng

Black ginseng is a more recent type of processed ginseng with a unique components profile, implying potent *in vitro* and *in vivo* pharmacological activities (Liu et al., 2010; Jin et al., 2015). A study showed that 2 weeks' black ginseng post-treatment improved the impairment of learning and memory in rats, indicated by the Morris water maze 2 weeks after MCAO (2 h) (Park et al., 2011).

### Ginseng Total Saponins

Ginsenosides (ginseng total saponins, GTS) may be mainly responsible for the pharmacological effects of ginseng. GTS treated rats have better neurological scores compared with those in control group at 14d after pMCAO (Zheng et al., 2011).

### Ginsenosides Rb and Ro Mixture

It was reported that pretreatment with ginsenosides Rb and Ro mixture (which was hard to purify due to similar polarity), markedly reduced ischemic brain edema in rats at 1 h following GCI (4VO) (Chu and Chen, 1990).

### Fermented Red Ginseng

Fermented red ginseng was reported to be prepared from red ginseng extract, and the primary components were compound K > ginsenoside Rg3 > or = ginsenoside Rh2 (Bae et al., 2004). It was shown to protect against ischemic brain injury, indicated by the significant reduction of infarct volume after 22 h of reperfusion.

## Neuroprotective Mechanisms of Panax Ginseng in Stroke

The discovery of the beneficial effects of ginseng or ginsenosides on ischemic and hemorrhagic stroke has spurred interest in their mechanisms of action. Multiple potential neuroprotective mechanisms were evaluated during the studies (Table 2).

**Table 2:** The putative neuprotective mechanisms of Panax ginseng in experimental stroke.

**Stroke model**	**Species**	**Genetic background**	**Type of ginseng extracts**	**Main mechanisms (*in vivo*)**	**Authors/year**
pdMCAO	Mouse	C57BL/6	Red ginseng (KRG)	• Nrf2 pathway • Oxidative stress • Reactive astrogliosis and microglia activation • Glutamine synthetase (GS), Aquaporin-4 (AQP4)	Liu et al., 2018b
MCAO (1 h)	Mouse	C57BL/6	Red ginseng (KRG)	• Oxidative stress (8-hydroxyguanosine) • Apoptosis signal-regulating kinase 1 (ASK1)	Cheon et al., 2013
MCAO (2 h)	Rat	SD	Red ginseng (KRG)	• Levels of lipid peroxidation	Ban et al., 2012
MCAO (2 h)	Rat	SD	Red ginseng (KRG)	• Inflammatory cytokines [tumor necrosis factor-alpha (TNF-α), interleukin-1 beta (IL-1β), and IL-6, IL-10]	Lee et al., 2011
Hypoxia-ischemia	Mouse	C57BL/6	Red ginseng (KRG)	• Nrf2 pathway • Oxidative stress • Neuroinflammation • Reactive astrogliosis and microglia activation • Glutamine synthetase (GS), Aquaporin-4 (AQP4)	Liu et al., 2019
pdMCAO	Rat	SHR-SP	Ginsenoside Rb1	• Upregulation of Bcl-x(L) expression • (activation of mitochondrial cell death signaling)	Zhang et al., 2006
MCAO (1 h)	Mouse	C57BL/6J	Ginsenoside Rb1	• oxidative stress • Extracellular signal-regulated Kinase (ERK) signaling activation	Dong et al., 2017
MCAO (1 h)	Mouse	ICR	Ginsenoside Rb1	• BBB (evans blue, ZO1, and occludin proteins) • Inflammation (iNOS, IL-1β, IL-10) • Oxidative stress	Chen et al., 2015
MCAO (1.5 h)	Rat	SD	Ginsenoside Rb1	• Autophagy [LC3 (I, II) and Beclin1 proteins]	Lu et al., 2011
MCAO (2 h)	Rat	SD	Ginsenoside Rb1	• Inflammation (IL-6, gene, and protein levels) • Nuclear factor-κB (NF-κB) pathway • (expression of total and phosphorylated NF-κB/p65, inhibitor protein of κB (IκB)-α, and IκB-kinase complex (IKK)-α)	Zhu et al., 2012
MCAO (2 h)	Rat	Wistar	Ginsenoside Rb1	• Modulations of apoptotic-related genes and glial-derived neurotrophic factor (GDNF) expression	Yuan et al., 2007
MCAO (2 h)	Rat	NA	Ginsenoside Rb1	• Neural cell apoptosis • Expressions of Bcl-2 and Bax	Yang et al., 2008
MCAO (2 h)	Rat	Wistar	Ginsenoside Rb1	• Brain-derived neurotrophic factor (BDNF) • Caspase-3 protein	Gao et al., 2010
MCAO (2 h)	Rat	Wistar	Ginsenoside Rb1	• BBB permeability • Aquaporin-4 (AQP4)	Li et al., 2018b
MCAO (2 h)	Rat	Wistar	Ginsenoside Rb1	• Inflammation [tumor necrosis factor-alpha (TNF-α), interleukin-6 (IL-6), iNOS, and NO] • High mobility group box 1 (HMGB1)	Liu et al., 2018a
MCAO (2 h)	Rat	SD	Ginsenoside Rb1	• IL-1 beta	Liu et al., 2013
GCI (2VO)	Rat	Wistar	Ginsenoside Rb1	• Augophagy (LC3II and Beclin1) • Phosphatidylinositol 3-kinase (PI3K)/Akt pathway	Luo et al., 2014
GCI (4VO)	Rat	NA	Ginsengoside Rb1	• Improve cerebral glucose utilization	Choi et al., 1996
SAH	Rat	NA	Ginsenoside Rb1	• Apoptosis (P53, Bax, and Caspase-3 proteins)	Li et al., 2010
pdMCAO	Rat	SH-SP	Ginsenoside Rb1	• ND	Zhang et al., 1998
SAH	Rat	SD	Ginsenoside Rb1	• ND	Li et al., 2011b
pMCAO	Rat	SD	Ginsenosides Rb1 and Rg1	• ND	Zhang and Liu, 1996
MCAO (1.5 h)	Rat	SD	Ginsenoside Rg1	• Oxidative stress [myeloperoxidase (MPO), superoxide dismutase (SOD), catalase (CAT)] activities • Inflammation (IL-6, TNFα) • Peroxisome proliferator-activated receptor γ (PPARγ), NF-κB	Li et al., 2017b
MCAO (2 h)	Mouse	C57BL/6J	Ginsenoside Rg1	• Inflammation (IL-1β, IL-6, and TNFα) • Excitatory amino acids such as the contents of Glu and Asp (by High-performance liquid chromatography)	Wang et al., 2018a
MCAO (2 h)	Rat	SD	Ginsenoside Rg1	• Apoptosis (TUNEL) • Extracellular signal-regulated kinase 1/2 (ERK1/2), phosphorylated extracellular signal-regulated kinase 1/2 (p-ERK1/2), c-Jun N-terminal kinases (JNK), and phosphorylated c-Jun N-terminal kinase (p-JNK)	Wang et al., 2013a
MCAO (2 h)	Rat	SD	Ginsenoside Rg1	• PPARγ/Heme oxygenase1 (HO1) signaling (suppress both apoptosis and inflammation) • PPARγ, bcl-2, cleaved caspase-3, cleaved caspase-9, IL-1β, TNF-α, High mobility group box 1 (HMGB1), and Receptor for advanced glycation end products (RAGE)	Yang et al., 2015b
MCAO (2 h)	Rat	SD	Ginsenoside Rg1	• Metabolic regulation	Lin et al., 2015
MCAO (2 h)	Rat	SD	Ginsenoside Rg1	• Ca^2+^ influx through NMDA receptors and L-type voltage-dependent Ca^2+^ channels	Zhang et al., 2008b
MCAO (2 h)	Rat	SD	Ginsenoside Rg1	• BBB integrity • Aquaporin 4	Zhou et al., 2014
MCAO (2 h)	Rat	SD	Ginsenoside Rg1	• BBB integrity • Regulation of protease-activated receptor-1 expression	Xie et al., 2015
MCAO (2 h)	Rat	SD	Ginsenoside Rg1	• BBB integrity • matrix metalloproteinases (MMPs)	Wang et al., 2013b
MCAO (1 h)	Mouse	BALB/c	Ginsenoside Rg1	∙ ND	Sun et al., 2014a
MCAO (1 h)	Mouse	C57BL/6	Ginsenoside Rd	• Mitochondrial dysfunction • Antioxidant activities	Ye et al., 2011b
MCAO (1.5 h)	Rat	SD	Ginsenoside Rd	• Neurogenesis	Liu et al., 2015
MCAO (2 h)	Rat	SD	Ginsenoside Rd	• Early oxidative damage and sequential inflammatory response (Free radical generation (microdialysis), oxidative DNA damage (8-hydroxy-deoxyguanosine), oxidative protein (protein carbonyl and advanced glycosylation end products), lipid peroxidation (the malondialdehyde and 4-hydroxynonenal formations)	Ye et al., 2011c
MCAO (2 h)	Rat	SD	Ginsenoside Rd	• Mitochondrial enzyme activities, mitochondrial membrane potential (MMP), production of ROS, energy metabolites, and apoptosis	Ye et al., 2011d
MCAO (2 h)	Rat	SD	Ginsenoside Rd	• AIF mitochondrio-nuclear translocation and NF-κB nuclear accumulation by inhibiting poly (ADP-ribose) polymerase-1	Hu et al., 2013
MCAO (2 h)	Rat	SD	Ginsenoside Rd	• Microglial activation • Pro-inflammatory Cytokines (IL-1β, IL-6, IL-18, TNFα, and IFN-γ) • Alpha (IκBα) phosphorylation and NF-κB nuclear translocation	Zhang et al., 2016
MCAO (2 h)	Rat	SD	Ginsenoside Rd	• Tau protein phosphorylation • PI3K/AKT/GSK-3β pathway	Zhang et al., 2014a
MCAO (2 h)	Rat	SD	Ginsenoside Rd	• Mitochondrial DNA (mtDNA) and nuclear DNA (nDNA) damages	Yang et al., 2016
MCAO (2 h)	Rat	SD	Ginsenoside Rd	• The phosphorylation of the NMDAR 2B subunit (NR2B subunit)	Xie et al., 2016
pMCAO	Rat	SD	Ginsenoside Rd	• ND	Ye et al., 2011a
MCAO (1.5 h)	Rat	SD	Ginsenoside Rd	• ND	Zhang et al., 2012
MCAO (2 h)	Rat	Wistar	Ginsenoside Re	• Oxidative stress [lipid peroxidation: malondiadehyde (MDA) formation], superoxide dismutase (SOD) and glutathion peroxidase (GSH-Px)]	Zhou et al., 2006
MCAO (2 h)	Rat	SD	Ginsenoside Re	• Oxidative stress (MDA)	Chen et al., 2008b
MCAO (2 h)	Rat	SD	Ginsenoside Rh2	• ND	Park et al., 2004
MCAO (2 h)	Rat	SD	20(R)-Ginsenoside Rg3	• Apoptosis (TUNEL) • Calpain I and caspase-3	He et al., 2012
MCAO (0.5 h)	Mouse	C57BL/6	Compound K	• Inflammation • Microglial activation	Park et al., 2012
MCAO (1.5 h)	Rat	SD	Black ginseng (produced from red ginseng)	• ND	Park et al., 2011
pMCAO	Rat	Wistar	Ginseng total saponins (GTS)	• Neurogenesis	Zheng et al., 2011
GCI (4VO)	Rat	Wistar	Panax Ginseng extracts	• Oxidative (lipid peroxidation: MDA, SOD and GPx)	Kim et al., 2009
GCI (4VO)	Rat	SD	Ginsenosides Rb + R0	• Oxidative stress [Anti-lipid peroxidation: creatine phosphokinase (CK) and SOD]	Chu and Chen, 1990

*MCAO, middle cerebral artery; pdMCAO, permanent distal middle cerebral artery occlusion; pMCAO, permanent (proximal end of) middle cerebral artery occlusion that is generated by the intraluminal suture MCAO; GCI, global cerebral ischemia; SAH, subarachnoid Hemorrhage; BBB, blood brain barrier; NA, no answer; ND, non-discussed*.

### Anti-oxidative Stress

Redox homeostasis in the cell is maintained by the counterbalance between reactive oxygen and nitrogen species (ROS/RNS) generation and the antioxidant defense system (Lin and Beal, 2006; Ma, 2013). Oxidative stress is a result of imbalance between the ROS/RNS and the antioxidant defense system. ROS/RNS are constantly produced by oxygen metabolism in accordance with the rate of oxidant formation and elimination, most of which comes from mitochondria (Balaban et al., 2005). Under normal conditions, only 1–2% of molecular oxygen is converted into superoxide radicals (Orrenius et al., 2007; Drummond et al., 2011) and then removed by the potent and extensive antioxidant system. However, under stress conditions like stroke attack, the overproduction of ROS and the reduced antioxidant capacity result in oxidative damage to DNA, RNA, lipids, and other cell components, eventually leading to cell death. The central nervous system (CNS) is typically vulnerable to oxidative stress as it consumes a higher amount of oxygen and has a lower level of endogenous antioxidant defense capacity than other organs (Sims and Muyderman, 2010; Chen et al., 2011; Sinha and Dabla, 2015). The rapid increase in the production of ROS/RNS immediately following stroke overwhelms the antioxidant defense system, damaging cellular macromolecules which leads to apoptosis, autophagy, and necrosis (Rodrigo et al., 2013). Moreover, the restoration of blood flow further increases the tissue oxygenation level and initiates a second burst of ROS/RNS overproduction, triggering reperfusion injuries (Sims and Muyderman, 2010; Rodrigo et al., 2013). Given that oxidative stress occurs early and acts causally in stroke pathogenesis (Chen et al., 2011), therapies targeting basic oxidative processes, such as free-radical generation or specific antioxidants that interact with stroke-related proteins, hold great promise (Becerra-Calixto and Cardona-Gomez, 2017; Bhatti et al., 2017).

Accumulated evidence demonstrated the beneficial efficacy of ginseng against various CNS diseases, mainly owing to its anti-oxidative and anti-inflammation properties (Gonzalez-Burgos et al., 2015; Ahmed et al., 2016; Lee et al., 2017). Several studies supported that, associated with their benefits on stroke outcomes, ginseng, or ginsenosides have the antioxidant potential against stroke damage by scavenging overproduced ROS/RNS via modulating endogenous antioxidant defense system. KRG attenuated the oxidative damages indicated by the reduced levels of 8-hydroxyguanosine (8-OHG) (Cheon et al., 2013), a biomarker of oxidative DNA damage, and lipid peroxidation (Ban et al., 2012) and the increased antioxidant related protein levels in superoxide dismutase 2 (SOD2), glutathione peroxidase 1 (Gpx1), heme oxygenase 1 (HO1), and NAD(P)H quinone dehydrogenase 1 (NQO1) (Liu et al., 2018b, 2019) compared to controls. Mitochondrial SOD2 is one critical component of the antioxidant system, accounting for the removal of superoxide ions in the mitochondria (Flynn and Melov, 2013). GPx is another key antioxidant enzyme that catalyzes the reduction of lipid peroxides and hydroperoxide to non-toxic species. Superoxide in the mitochondrial matrix is metabolized to hydrogen peroxides by SOD2 and decomposed to water by GPx (Ghosh et al., 2011). HO1, an inducible enzyme, has emerged as a major protective mechanism against oxidative stress (Zeynalov et al., 2009). In addition, these findings above are also supported by the results in GCI model (Luo et al., 2014). Rb1 was shown to have neuroprotective effects on brain damage by anti-oxidant activity, indicated by the levels of glutathione (GSH), MDA, nitric oxide (NO), nicotinamide adenine dinucleotide phosphate (NADPH) oxidase (NOX) expression and NADPH oxidase activity (Dong et al., 2017). Rg1 increased the activity or content of antioxidant enzymes SOD and catalase (CAT) (Li et al., 2017b), as well as HO1 (Yang et al., 2015b), contributing to the histological and functional benefits after stroke. Rd treated animals exhibited a reduced level in free radical generation revealed by microdialysis, oxidative DNA damage (8-OHG), oxidative proteins carbonyl and advanced glycosylation end products (AGEs), lipid peroxidation [malondialdehyde (MDA) and 4-hydroxynonenal formations (4-HNE)] following MCAO (Ye et al., 2011c). Rd administration also reduced mitochondrial DNA (mtDNA) and nuclear DNA (nDNA) damages, which contributed to an improvement in survival rate and neurological function (Yang et al., 2016). It was observed that Re significantly ameliorated lipid peroxidation by raising the activities of SOD and GSH-Px, and reduced the content of MDA in rat brains protecting against cerebral ischemia-reperfusion injury (Zhou et al., 2006), which was supported by another Re study in MCAO (Chen et al., 2008b).

In recent years, fundamental progress in the oxidative stress research field was the discovery of transcriptional factor Nrf2/antioxidant response element (ARE) pathway, which is the master regulator of redox hemostasis by tightly controlling multiple ARE-driven antioxidant proteins likeNQO1 and HO1 (Cuadrado et al., 2018; Raghunath et al., 2018; Yamamoto et al., 2018). In response to stress conditions or Nrf2 inducers, Nrf2 protein is liberated from Kelch-like ECH-associating protein 1 (Keap1)-mediated repression, translocates into the nucleus, binds to the ARE sequence in the promoter region of Nrf2 target proteins, thereby activating a wide range of cytoprotective genes (Hayes and Dinkova-Kostova, 2014). Very recently, pretreatment with KRG, as an Nrf2 inducer, significantly increased the expression levels of Nrf2 target cytoprotective and antioxidant proteins after pdMCAO, which was abolished in ischemic-Nrf2^−/−^ mice, supporting the Nrf2-dependent neuroprotection of KRG in ischemic stroke (Liu et al., 2018b). This is supported by other *in vivo* (Yang et al., 2015b; Gao et al., 2017a; Li et al., 2017a) and *in vitro* (Hwang and Jeong, 2010) reports. In addition, pretreatment of ginsenoside Rb1 was reported to have anti-oxidant neuroprotective effects through promoting ERK1/2 pathways in cerebral ischemia-induced injuries in aged mice (Dong et al., 2017).

Astrocytes are recognized to exert essential and complex functions for maintaining normal neural activity in the healthy CNS and respond to various forms of CNS injury or disease. Reactive astrogliosis, regulated in a context specific manner, alters astrocytic functions and thereby exerts beneficial effects on neural functions. Given the important role of astrocytes in oxidative stress and inflammation process (Hamby and Sofroniew, 2010; Sofroniew, 2014; Ong et al., 2015), reactive astrogliosis was considered to contribute to the neuroprotection of ginseng in stroke. Indeed, in permanent cerebral ischemia model mice, ginseng pretreatment robustly attenuated the acute reactive astrogliosis progression but not the microglia activation in the ischemic cortex region in an Nrf2-dependent manner. The spatial and temporal pattern correlated well with the acute ischemic damage expansion during the acute stage of ischemia (Liu et al., 2018b). In addition, ginseng pretreatment was found to attenuate the deterioration of glutamine synthetase, the key enzyme for glutamate metabolism, and aquaporin 4 (AQP4), the unique water channel that is predominantly distributed in astrocytes. One of the major causes of morbidity and mortality after stroke is brain edema; the influence of ginseng on cellular water penetrability at least partly involves its favorable effects on stroke damage. In agreement with this observation, it was reported that the neuroprotection of Rg1 against ischemic-reperfusion brain injury might be associated with the reduced expression AQP4 level (Zhou et al., 2014).

### Anti-inflammation

Inflammation is another major player that is involved in stroke pathogenesis, which contributes to all the stages of the stroke pathophysiology (Iadecola and Anrather, 2011; Fu et al., 2015; Esenwa and Elkind, 2016; de Oliveira Manoel and Macdonald, 2018; Drieu et al., 2018). The inflammatory responses are typically mediated by pro-inflammatory prostaglandins, cytokines and chemokines. These components attract immune cells, interact with the adaptive immune system, and evoke the systemic release of acute phase reactants (Esenwa and Elkind, 2016). These pro-inflammation proteins include IL-1β, IL-6, tumor necrosis factor α (TNFα), interferon γ (IFNγ), complement proteins, C-reactive protein (CRP), etc., which are implicated in the pathogenesis and progression of atherosclerosis and intravascular thrombosis (Sofroniew, 2015; Drieu et al., 2018). Anti-inflammatory mediators include IL-4, IL-10, TGFβ, etc. (Mandolesi et al., 2015; Sofroniew, 2015). Microglial activation plays an important role in inflammation, and activated microglia have both pro- and anti-inflammatory properties (Hoogland et al., 2015).

Anti-inflammation might be another intriguing neuroprotective effect of ginseng. Suppression of inflammation contributed to the neuroprotection of Rb1 on cerebral ischemic injury and the integrity of blood-brain barrier (BBB), indicated by the downregulated expression of pro-inflammatory factors nitric oxide synthase, IL-1β, IL-6, and upregulated expression of anti-inflammatory markers arginase 1 and IL-10 in the ischemic brain (Zhu et al., 2012; Chen et al., 2015). Rg1 was reported to suppress inflammation and preserve the brain tissue from stroke insults (Wang et al., 2018a), and the underlying mechanism was related to the activation of PPARγ/HO-1 (Yang et al., 2015b) and PPARγ-regulated pathways (Li et al., 2017b). The beneficial effect in inhibition of inflammation was also observed in MCAO rats treated with KRG (Ban et al., 2012) or Rd (Zhang et al., 2016). In addition, Rb1 and Rd have been shown to repress microglial activation and decrease the pro-inflammatory cytokines IL-6 in a transientMCAO rat model, which resulted in a decrease in infarct volume and neurological deficits score (Ye et al., 2011c; Zhu et al., 2012).

Nuclear factor-κB (NF-κB) is a critical transcription factor involved in the regulation of inflammation through the target genes such as cycloygenase-2 (COX-2), inducible nitric oxide synthase (iNOS), and IL-6 (Harari and Liao, 2010). A study showed that Rb1 can suppress NF-κB and its DNA binding activity thus suppressing neuronal death as well as decreasing IL-6 levels in the brain with cerebral ischemia (Zhu et al., 2012). Rg1 was shown to exert its neuroprotective action through antioxidative and anti-inflammatory effects mediated by the activation of PPARγ signaling, and the beneficial effect was abolished by a selective PPARγ antagonist GW9662 (Li et al., 2017b). The administration of Rd after stroke inhibited ischemia-induced microglial activation, decreased the expression levels of various proinflammatory cytokines, and suppressed nuclear factor of kappa light polypeptide gene enhancer in B cells inhibitor, alpha (IκBα) phosphorylation and NF-κB nuclear translocation (Zhang et al., 2016). The anti-inflammatory effect of Rd was also supported by another report. It was shown that Rd significantly eliminated inflammatory injury as indicated by the suppression of microglial activation and reduced pro-inflammatory factors levels (Ye et al., 2011c). Mitogen-activated protein kinases (MAPKs) mediate another group of signal transduction pathways activated by stress and inflammation that enhance the formation of pro-inflammatory proteins in stroke. The p38 MAPK can downregulate HO1 expression, which has potent anti-inflammatory, antioxidant and anti-apoptotic properties (Naidu et al., 2009; Jang et al., 2012; Wang et al., 2015). Compound K showed a neuroprotective effect on experimental stroke in mice through inhibiting phosphorylation of MAPKs and enhancing HO1 expression, thus decreasing production of pro-inflammatory proteins in activated microglia (Park et al., 2012).

### Anti-apoptosis

Another important role of ginseng on ischemic stroke is the inhibition of apoptosis or cell death. There is a dynamic balance between anti-apoptotic proteins (such as Bcl-2) and pro-apoptotic proteins [such as Bcl-2-associated X protein (Bax)], playing a major role in regulating apoptosis. Rb1 was shown to increase Bcl-2 protein and decrease BAX protein in MCAO model rats (Yuan et al., 2007). Similarly, Rg2 was shown to increase Bcl-2 protein and decrease Bax protein in rats after MCAO (Zhang et al., 2008b). Bcl-2 is mainly located in the mitochondrial outer membrane, and cytochrome c, a small heme protein, is mainly located in the mitochondrial inner membrane, signifying the important role of mitochondria in apoptosis process. Rd was reported to attenuate mitochondrial release of AIF, caspase 3 and cytochrome c in MCAO rats, leading to the benefit of Rd on ischemic brain lesion (Ye et al., 2011d). Rb1 can also decrease the activity of caspase 3 in the ischemic brain of rats, thus inhibiting cell death after MCAO (Gao et al., 2010). KRG extract decreased the number of apoptosis signal-regulating kinase 1 (ASK1)-positive cells and the expression level of ASK1 protein in the ischemic region at 4 and 24 h after MCAO, resulting a better performance in ischemic rats (Cheon et al., 2013).

### Anti-autophagy

Autophagy is a self-eating cellular catabolic pathway, degrading and recycling damaged organelles and misfolded proteins for cellular homeostasis (Wang et al., 2018b). Due to its important homeostatic role in regulating cell survival, emerging evidence showed that autophagy is implicated in the destructive process in stroke (Wu et al., 2016; Li et al., 2018a). LC3, a crucial protein for autophagy, is mainly located in the cytoplasm and concentrated in autophagosomes during autophagy. Beclin1 also plays a key role in the regulation of autophagosome formation. In MCAO model rats, Rb1 attenuated autophagy via a decrease in the associated proteins LC3 and Beclin 1 in transient MCAO rat models (Lu et al., 2011). In GCI (2VO) model rats, Rb1 administration inhibited autophagy in hippocampal CA1 neurons, evidenced by the expression level of autophagy hallmark proteins LC3 (I and II) and Beclin1 in CA1 neurons by confocal microscopy and Western blot (Luo et al., 2014).

### Other Beneficial Mechanisms

Stroke is a heterogeneous and multi-factorial cerebrovascular disease; multiple cell death pathways are evoked in response to acute brain injury (Kellner and Connolly, 2010; Fisher, 2011; Tasker and Duncan, 2015). Such injury induces various endogenous protective mechanisms, including neurogenesis, angiogenesis, and vascular remodeling responses (Marti and Risau, 1999; Greenberg, 2014; Seto et al., 2016; Koh and Park, 2017). To enhance the endogenous neurogenesis driven by ischemia and promote the survival of newborn neurons are considered as the promising therapeutic interventions for stroke (Lu et al., 2017). It was shown that GST and Rb1 increased the numbers of neuronal precursors and promoted the proliferation of endogenous neural stem cells, thus promoting the behavior recovery post-ischemia (Gao et al., 2010; Zheng et al., 2011). Re was shown to improve the fluidity of the mitochondrial membrane that was important for energy generation (Zhou et al., 2006).

Angiogenesis refers to the process of new blood vessel formation from the existing vasculature (Adair and Montani, 2010). Although the vascular system in the adult brain is extremely stable under normal conditions, pathological angiogenesis is induced in response to brain ischemia. The angiogenesis induction, mainly in the ischemic area, enhances the supply of oxygen and nutrients. Therefore, post-stroke angiogenesis facilitates the process of vascular remodeling and is considered a harmonized target for neurological recovery (Mennel, 2000; Beck and Plate, 2009; Dejana, 2010; Xiong et al., 2010; Ergul et al., 2012). The angiogenic factors are induced within hours following stroke, and new capillaries are developed within days (Greenberg, 2014). Ginsengosides have indicated salutary effects on angiogenesis in stroke through inducing various angiogenesis regulators. Ginsenoside Rg1 was shown to facilitate angiogenesis after hypoxia/ischemia brain injury, and the pharmacological effects of Rg1 may be attributed to the regulation of the vascular endothelial growth factor (VEGF) and cleaved caspase 3 expression levels (Tang et al., 2017). Ginsenoside Rg1 was also reported to improve angiogenesis in the diabetic ischemic hind limb, and the potential mechanism might be related to the eNOS activation and upregulation of the VEGF expression (Yang et al., 2012).

## Translational Potential of Ginseng and Ginsenosides in Stroke Therapeutics

Since 1996 till now, one strategy for improving functional recovery after ischemic stroke is to restore blood flow to salvage ischemic tissue by introducing intravenous recombinant tissue plasminogen activator (rtPA) in acute ischemic stroke, while the other protocol if removal of the blood clot by thrombectomy (Prabhakaran et al., 2015; Romano and Sacco, 2015). Despite that only <40% patients who are treated with rtPA alone regain functional independence (Saver et al., 2015), more than 95% of patients receive only supportive care without rtPA treatment due to the narrow therapeutic window (up to 4.5 h) and limited indications (Hacke et al., 2008; Fonarow et al., 2011; Sandercock et al., 2012; Emberson et al., 2014). The other strategy is neuroprotection targeting various components of the cascade during ischemic insult, which is supported by preclinical data for many agents (Fisher, 2011; Dirnagl and Endres, 2014; Fisher and Saver, 2015). Unfortunately, all prior drug development of neuroprotective agents has been unsuccessful, no neuroprotective drug demonstrated unequivocal efficacy in clinical trials (Fisher, 2006; Hossmann, 2006; Della-Morte et al., 2013).

Many single-target stroke intervention strategies have failed to provide efficacy in clinical trials. The field is in tremendous need of new targets that exert pleiotropic effects on cellular viability through multiple mechanisms. Interestingly, ginseng could be beneficial for the prevention or treatment of stroke through regulating multipronged mechanisms that can provide the brain/cells with resistance against acute and chronic debilitating neurodegenerative conditions. Living organisms are continuously threatened by the damage caused by free radicals produced during normal oxygen metabolism and mitochondrial function or generated by exogenous damage. For centuries, ginseng has been reported as a preventive medicine capable of boosting the nervous system, but the effects on stroke and the underlying cellular mechanisms are still unclear. Increasing *in vivo* pre-clinical stroke studies of either pretreatment or posttreatment will provide a better understanding of the unique properties of ginseng and its derivatives in the preventive and therapeutic treatment of stroke.

## Concluding Remarks

The promising preventive and therapeutic efficacy of ginseng or ginsenosides on experimental stroke damage has been illuminated during the last decade. The putative neuroprotective mechanisms of ginseng or ginsenosides include anti-oxidant, anti-inflammation, anti-apoptosis, anti-autophagy, neurogenesis, and others. These effects have the potential to influence short- and long-term complex neurobehaviors such as the overall deficits, motor, sensorimotor, and cognition. It is known that stroke injury results in severe motor, sensory, emotional, and cognitive deficits (Ferro et al., 2016), and long-term functional recovery is considered as the ultimate goal of stroke intervention. Accordingly, more effective and long-term histological and neurological assessments are expected for future preclinical stroke studies. In addition, the responses to various forms of stroke insults involve complicated interactions among brain cells with numerous functions and lineages, including intrinsic neural cells, intrinsic non-neural cells, and extrinsic cells that come from the circulation. The contributions of different non-neuronal cell types to the progress after acute brain injury are of robust interests for future studies as the impetus toward understanding and ameliorating stroke insults.

## Review Criteria

We searched the PubMed and Embase databases by Jan 31, 2019 for the following terms individually or in combination: “ginseng,” “ginsenoside,” “stroke,” “ischemi^*^,” “ischaemi^*^,” “hemorrhage,” “hemorrhagic,” “subarachnoid,” “mouse,” “rat,” and their abbreviations. Study selection for inclusion and exclusion was performed based on predefined criteria. Selection of articles: (1) The studies were published in English; (2) The study clearly described the stroke model and administration route of ginseng or ginsenosides; (3) Ginseng or ginsenosides were administrated without the combination of other compounds. (4) The study was an original full paper that presented the data. Totally 402 articles in PubMed and 454 articles in Embase were identified. After screening analysis in title, abstract and full text and duplication analysis, 54 articles met inclusion criteria. Three independent investigators reviewed articles and extracted data for study design elements, such as animals, animal models, administration strategies, stroke outcomes, and mechanisms. We specifically focus on the *in vivo* evidence for the effects of ginseng and ginsenosides on various stroke damages and mechanism.

## Author Contributions

LL and SD conceived the study, designed the databases analysis, and wrote the manuscript. LL, GA, and TF searched databases, collected data, performed analyses, and prepared the tables and figure. All authors discussed and approved the final manuscript.

### Conflict of Interest Statement

The authors declare that the research was conducted in the absence of any commercial or financial relationships that could be construed as a potential conflict of interest.
